# Age‐related changes in miR‐143‐3p:Igfbp5 interactions affect muscle regeneration

**DOI:** 10.1111/acel.12442

**Published:** 2016-01-13

**Authors:** Ana Soriano‐Arroquia, Rachel McCormick, Andrew P. Molloy, Anne McArdle, Katarzyna Goljanek‐Whysall

**Affiliations:** ^1^ Institute of Ageing and Chronic DiseaseUniversity of Liverpool6 West Derby StreetLiverpoolL7 8TXUK; ^2^ University Hospital AintreeLiverpoolUK

**Keywords:** aging, microRNA, muscle, regeneration, sarcopenia, senescence

## Abstract

A common characteristic of aging is defective regeneration of skeletal muscle. The molecular pathways underlying age‐related decline in muscle regenerative potential remain elusive. microRNAs are novel gene regulators controlling development and homeostasis and the regeneration of most tissues, including skeletal muscle. Here, we use satellite cells and primary myoblasts from mice and humans and an *in vitro* regeneration model, to show that disrupted expression of microRNA‐143‐3p and its target gene, Igfbp5, plays an important role in muscle regeneration *in vitro*. We identified miR‐143 as a regulator of the insulin growth factor‐binding protein 5 (Igfbp5) in primary myoblasts and show that the expression of miR‐143 and its target gene is disrupted in satellite cells from old mice. Moreover, we show that downregulation of miR‐143 during aging may act as a compensatory mechanism aiming at improving myogenesis efficiency; however, concomitant upregulation of miR‐143 target gene, Igfbp5, is associated with increased cell senescence, thus affecting myogenesis. Our data demonstrate that dysregulation of miR‐143‐3p:Igfbp5 interactions in satellite cells with age may be responsible for age‐related changes in satellite cell function.

## Introduction

Age‐related loss of skeletal muscle mass and function results in frailty, decline in strength and decrease in quality of life of older people. Age‐related changes in muscle are thought to depend on a number of changes including mitochondrial production of reactive oxygen species (Jackson & McArdle, 2011), changes in muscle niche (Carlson & Conboy, 2007) and intracellular changes, such as changes in gene expression (Jejurikar *et al*., 2006). Regeneration of adult skeletal muscle is largely dependent on satellite cell population (Morgan & Partridge, 2003). The availability and functionality of satellite cells determine effective regeneration. Aging‐related changes in satellite cell number and properties, such as susceptibility to apoptosis and ability to proliferate, have been shown in humans and rodents (Brack *et al*., 2007; Collins *et al*., 2007; Verdijk *et al*., 2007). Moreover, the presence of senescent satellite cells in old mice and humans characterized by increased expression p16^INK4a^ and decreased phosphorylation of the retinoblastoma (RB) protein was shown (Sousa‐Victor *et al*., 2014).

microRNAs (miRNAs, miRs) affect the mRNA and protein content of the cell providing a rapid, high‐throughput mechanism of post‐transcriptional response to changes within and outside the cell. miRNAs are short, noncoding RNAs that regulate gene expression at the post‐transcriptional level. They are implicated in many biological processes, including skeletal muscle homeostasis (Goljanek‐Whysall *et al*., 2012b). miRs guide RNA‐induced silencing complex (RISC) to partially complementary sequences, usually contained within the 3′UTR of target mRNAs. miR interaction with its target(s) results in a translational block and often degradation of the mRNA. Occasionally, miRs activate the expression of their target. Muscle‐specific miRs are crucial regulators of skeletal muscle function (McCarthy & Esser, 2007; Goljanek‐Whysall *et al*., 2011, 2012a, 2014; Brown & Goljanek‐Whysall, 2015). Satellite cell‐specific Dicer knockout in a mouse model resulted in mild myofibre atrophy (Cheung *et al*., 2012). Although differential expression of miRs in muscle has been described during aging (Drummond *et al*., 2008; Kim *et al*., 2014), the relevance of these changes is not yet understood.

miR‐143‐3p (miR‐143) has an established role in the function of smooth muscle myoblasts and adipogenesis (Esau *et al*., 2004; Cordes *et al*., 2009; Elia *et al*., 2009; Xin *et al*., 2009). miR‐143‐5p is highly expressed in skeletal muscle tissue, and its expression is downregulated during aging (Kim *et al*., 2014). A set of miR‐143 predicted targets is associated with insulin‐like growth factor (IGF) signalling (Blumensatt *et al*., 2014). The IGF signalling pathway plays a crucial role in the maintenance of skeletal muscle homeostasis. IGF1 and IGF2 stimulate myoblast proliferation and differentiation, and these actions are mediated by the action of IGF1 receptor (IGF1R) (Duan *et al*., 2010). In addition to IGF1 receptors, IGF‐binding proteins (IGFBPs) are an important part of IGF signalling. IGFBPs are a family of proteins that bind IGF1 and IGF2 (Duan *et al*., 2010). IGFBPs not only regulate the half‐life of circulating IGF and modulate IGF activity in local tissues, but also have IGF‐independent functions (James *et al*., 1996; Cobb *et al*., 2004; Salih *et al*., 2004). The role of IGFBP5 in muscle is not fully understood; however, it has been shown to regulate myoblast proliferation, viability and differentiation, both via and independently of the IGF signalling (James *et al*., 1996; Cobb *et al*., 2004; Ren *et al*., 2008).

For the first time, this study investigates the regulation of IGF signalling‐related factor – Igfbp5 – by miR‐143 in satellite cells and the role of miR‐143:Igfbp5 interactions in myoblast viability, senescence and myogenic differentiation potential. We hypothesize that age‐related downregulation of miR‐143 expression and a concomitant upregulation of its target gene, Igfbp5, in satellite cells may act as a compensatory mechanism aimed to improve muscle regeneration during aging.

## Results

### miR‐143 is downregulated in satellite cells with age

**Figure 1:**
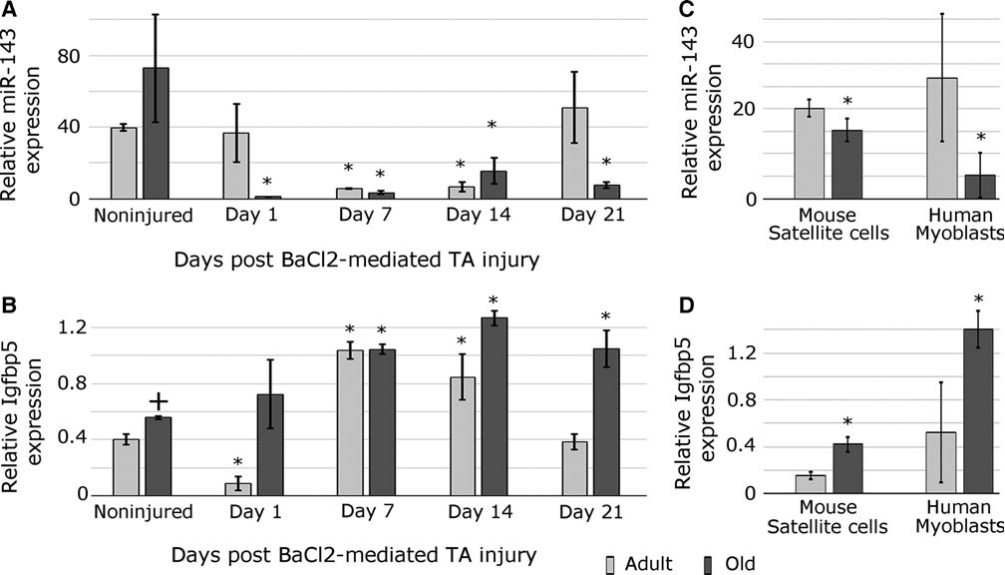
Expression of miR‐143 and Igfbp5 changes during aging and muscle regeneration. (A) qPCR demonstrating changes in miR‐143 expression following mouse muscle (TA, tibialis anterior) injury by barium chloride in the adult and old mice; day 0 – noninjured muscle; day 1/7/14/21 – days postinjury. (B) qPCR showing changes in Igfbp5 expression in TA muscle of the adult and old mice following injury. (C) qPCR showing the expression of miR‐143 in mouse satellite cells and human myoblasts during aging. (D) qPCR showing increased Igfbp5 expression in mouse satellite cells and human primary myoblasts during aging. Error bars show SEM, * – *P* < 0.05 (compared to adult or noninjured control, respectively), + – *P* < 0.05 (compared to adult); *n* = 3–4 biological replicates. Expression relative to Rnu‐6 (miR‐143) or β‐2‐microglobulin (Igfbp5) is shown.

To investigate whether miR‐143 may play a role during muscle regeneration, miR‐143 expression was analysed by qPCR during muscle regeneration (Fig. 1A). Following the injury of muscle of adult mice, the expression of miR‐143 was downregulated at days 7 and 14 postinjury and returned to the basal levels 21 days after injury (Fig. 1A). The expression of miR‐143 in the muscle of old mice following injury was downregulated at day 1 postinjury and remained low 21 days postinjury (Fig. 1A). This suggests that miR‐143 dysregulation of expression is relevant in regenerating muscle of old mice. To identify biologically relevant miR‐143 targets, candidate targets predicted by TargetScan Human (v 6.2) and with known functions in the muscle were selected for further analysis. This led to the identification of biologically relevant targets: Igf1r, Igfr2 and Igfbp5 – members of the IGF signalling (Fig. S3A). The expression of Igfbp5 mRNA was initially decreased (day 1) and next increased (from day 7) during the regeneration of muscle of adult mice and increased throughout the regeneration of muscle of old mice (Fig. 1B). The expression of Igf1r, but not Igf2r, during muscle regeneration also negatively correlated with the expression of miR‐143 (Fig. S2D).

As satellite cells play a key role during muscle regeneration, satellite cells from the muscle of adult and old mice were isolated by FACS (Fig. S1). Human primary myoblasts and not satellite cells were isolated due to the small size of the muscle sample. qPCR analysis demonstrated decreased expression of miR‐143 (Fig. 1C) and upregulated expression of Igfbp5 (Fig. 1D) in the satellite cells from old mice and primary myoblasts from older mice and humans. The expression of Igf1r, but not Igf2r, was also upregulated in the myoblasts from old mice and humans, but not in the satellite cells from old mice (Fig. S2C, S6E).

miR‐143 expression and Igfbp5, but not Igf1r, expression were inversely correlated in the satellite cells during aging (Fig. 1D, S6E), suggesting that miR‐143 may play a role in age‐related defective muscle regeneration through regulating the expression of Igfbp5.

### miR‐143 directly regulates the expression of Igfbp5

**Figure 2:**
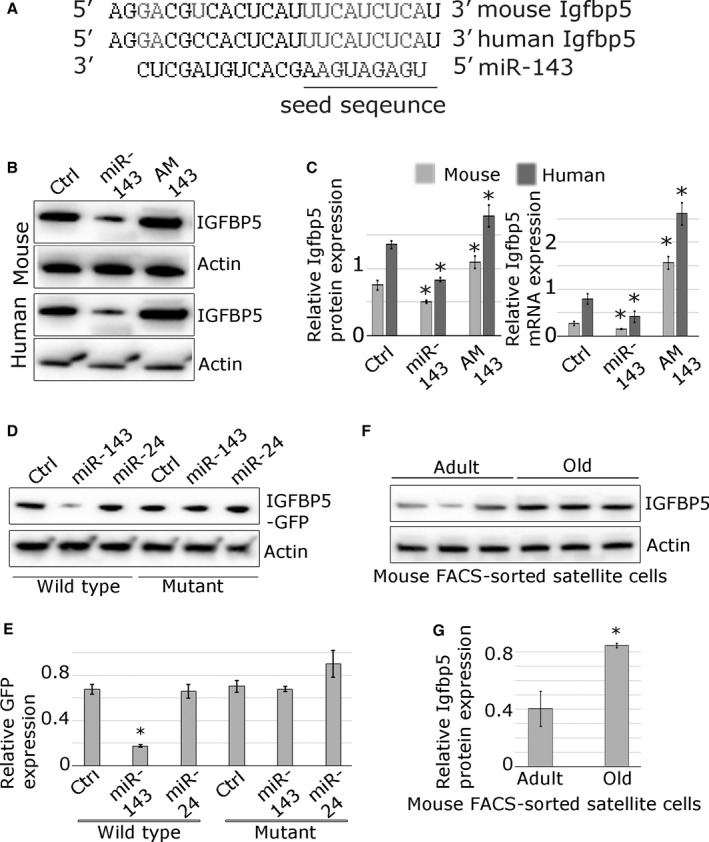
miR‐143 represses Igfbp5 expression in mouse and human primary myoblasts. (A) Alignment of putative miR‐143 target site in the 3′UTR of Igfbp5 gene; human and mouse sequences are indicated; conserved miR‐143 putative target site is indicated in grey; complementary nucleotides are shown in grey, and miR‐143 seed sequence is highlighted. (B, C) Endogenous Igfbp5 protein and mRNA expression is regulated by miR‐143 in the mouse and human myoblasts, as shown by Western blot or qPCR, respectively. (D, E) GFP‐Igfbp5 3′UTR sensor constructs containing conserved mouse wild‐type or mutated miR‐143 target site were transfected into mouse myoblasts. Co‐transfection with miR‐143 mimic (miR‐143), but not miR‐24 mimic (not predicted to target Igfbp5), led to the downregulation of GFP protein expression compared to mock‐transfected control (Ctrl), as shown by Western blot. Point mutations in the microRNA target site (mutant) rendered the sensor construct unresponsive. (F, G) Western blot showing increased expression of IGFBP5 protein in FACS‐sorted satellite cells from the old mice compared with the satellite cells from the adult mice. qPCR data show SEM; * – *P* < 0.05 (compared to control); representative Western blots are shown; *n* = 3.

The 3′UTR of Igfbp5 has one putative miR‐143 binding site conserved between humans and mouse (Fig. 2A). To validate Igfbp5 as physiologically relevant miR‐143 target, we investigated the expression of Igfbp5 in primary mouse and human myoblasts following miR‐143 overexpression or inhibition using miRNA mimic or anti‐miR (AM143), respectively (Fig. 2B, C). The efficiency of the transfections was validated in myoblasts (Fig. S3B, C, D). The expression of Igfbp5 mRNA and protein in mouse and human myoblasts was downregulated following the overexpression of miR‐143 and upregulated following the inhibition of miR‐143 function in primary myoblasts (Fig. 2B, C). To establish whether miR‐143 directly interacts with Igfbp5 3′UTR, we generated a reporter containing a fragment of Igfbp5 3′UTR downstream of GFP reporter (‘wild‐type’, WT). ‘Mutant’ reporter contained mutated miR‐143 binding site. Myoblasts were co‐transfected with miR‐143 mimic or miR‐24 mimic, not predicted to target Igfbp5. The GFP reporter containing WT Igfbp5 3′UTR was efficiently regulated by miR‐143, but not by miR‐24 (Fig. 2D, E). Mutation of the putative target site in the 3′UTR rendered the reporter insensitive to miR‐143, indicating that interaction with the target site is required for the response (Fig. 2D, E). To further demonstrate the biological relevance of miR‐143 targeting Igfbp5, we analysed IGFBP5 expression in FACS‐sorted satellite cells from the adult and old mice (Fig. 2F, G). As anticipated, IGFBP5 protein expression was inversely correlated with the expression of miR‐143 in the satellite cells from both adult and old mice.

### miR‐143 regulates myogenesis in an *in vitro* model of muscle regeneration

**Figure 3:**
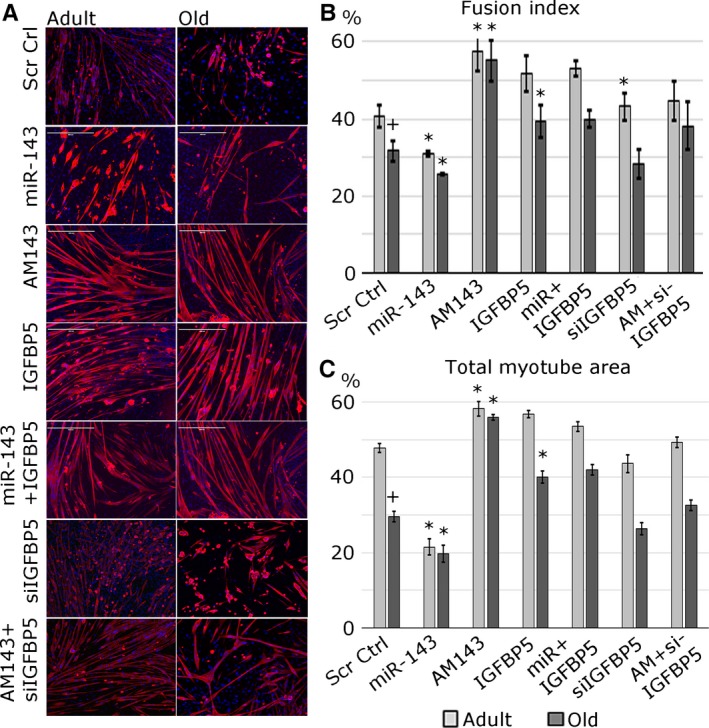
miR‐143 negatively regulates the regeneration of muscle *in vitro* via repressing Igfbp5 expression. (A) Satellite cells migrating out of isolated single myofibres were transfected with miR‐143 mimic (miR‐143), anti‐miR (AM143) and/or Igfbp5 overexpression construct or siRNA against Igfbp5, as indicated; new myotube formation was established by myosin heavy chain immunostaining: MF20 – red; DAPI – blue. (B) Fusion index is shown as % of nuclei contained within myotubes to the total number of myonuclei in each microscopic field. (C) Quantification of total myotube area is shown. Error bars show SEM, *, + – *P* < 0.05 (* – compared to Ctrl; + – compared to adult Ctrl), *n* = 3–4.

To validate the role of miR‐143:Igfbp5 interactions in a physiologically relevant context, an *in vitro* model of muscle regeneration was used (Fig. 3). The changes in expression levels of miR‐143 and Igfbp5 following transfections were physiologically relevant (5‐ to 10‐fold; Fig. S3B, C). The potential of satellite cells to form new myotubes (‘regenerate’) was established by quantifying the total myotube area and fusion index (Fig. 3). Satellite cells from the adult mice formed more myotubes containing more nuclei as compared with the satellite cells from the old mice (Fig. 3). miR‐143 overexpression led to the formation of fewer myotubes (Fig. 3A, C) containing less nuclei (Fig. 3A, B) compared with mock‐transfected cells. Conversely, miR‐143 inhibition led to the formation of more myotubes (Fig. 3A, C) containing more nuclei (Fig. 3A, B) compared with control. Overexpression of IGFBP5 in the satellite cells from the old mice resulted in the formation of a higher number of myotubes and higher fusion index (Fig. 3). Interestingly, upregulation of IGFBP5 expression rescued the effect related to miR‐143 overexpression and silencing of Igfbp5 abolished the effect of miR‐143 inhibition – the myotubes formed were characterized by fusion index similar to the control sample (Fig. 3). These data suggest that miR‐143:Igfpb5 interactions play a biologically relevant role during myogenesis *in vitro*.

### miR‐143 regulates myogenic differentiation *in vitro*

**Figure 4:**
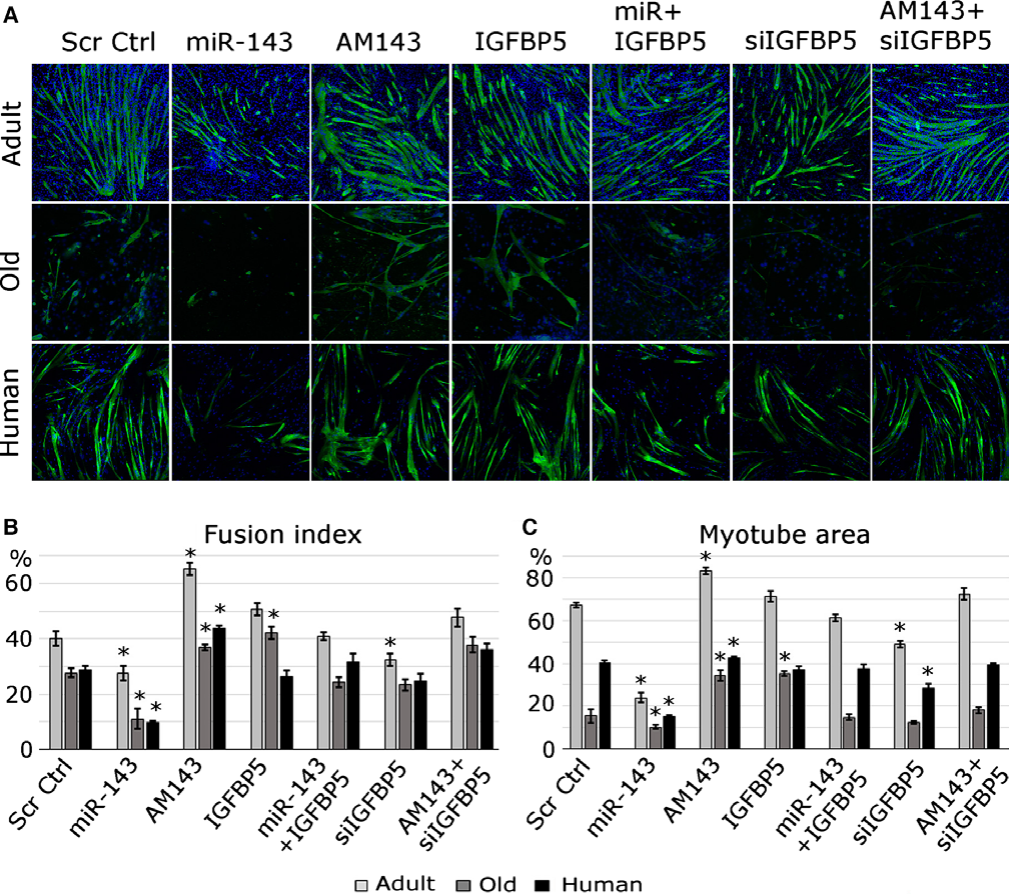
miR‐143 negatively regulates the differentiation of mouse and human myoblasts. Expression of miR‐143 and its target gene, Igfbp5, was manipulated in undifferentiated myoblasts using miR‐143 mimic (miR‐143), anti‐miR‐143 (AM143), Igfbp5 overexpression construct (IGFBP5) and/or siRNA against Igfbp5; myotubes were stained for myosin heavy chain: MF20 ‐ green; blue – DAPI; adult – 7‐month‐old mice; old – 24‐month‐old mice; human – adult. (A) MF20 immunostaining showing myogenic differentiation regulation by miR‐143. (B) Quantification of fusion index is shown. (C) Quantification of total myotube area is shown (%). Error bars show SEM, * – *P* < 0.05 (compared to control), *n* = 3–4.

Efficient muscle regeneration depends on activation, proliferation and viability of satellite cells, as well as their differentiation potential. miR‐143 gain‐ and loss‐of‐function approaches were used to establish the role of miR‐143 in myogenic differentiation *in vitro*. miR‐143 overexpression in mouse and human myoblasts resulted in the formation of fewer myotubes containing less nuclei, whereas the inhibition of miR‐143 led to the formation of more myotubes containing more nuclei, as shown by total myotube area and fusion index (Fig. 4). Overexpression of IGFBP5 in the myoblasts from the old mice resulted in the formation of more myotubes with a significant change in fusion index (Fig. 4). Silencing of IGFBP5 expression resulted in a decreased fusion efficiency of myoblasts from the adult, but not old, mice. Co‐transfection of miR‐143 with IGFBP5 overexpression vector or AM143 with siRNA against Igfbp5 resulted in rescuing of the phenotype associated with miR‐143 overexpression or inhibition, respectively (Fig. 4). Interestingly, the effects of miR‐143 or IGFBP5 expression manipulation were consistent during terminal differentiation (Fig. S4). These data suggest that miR‐143:Igfbp5 interactions may play a role in regulating the efficiency of myogenic differentiation, with miR‐143, IGFBP5 and potentially other miR‐143 targets, controlling myoblasts fusion.

### miR‐143:Igfbp5 interactions regulate myoblast viability

**Figure 5:**
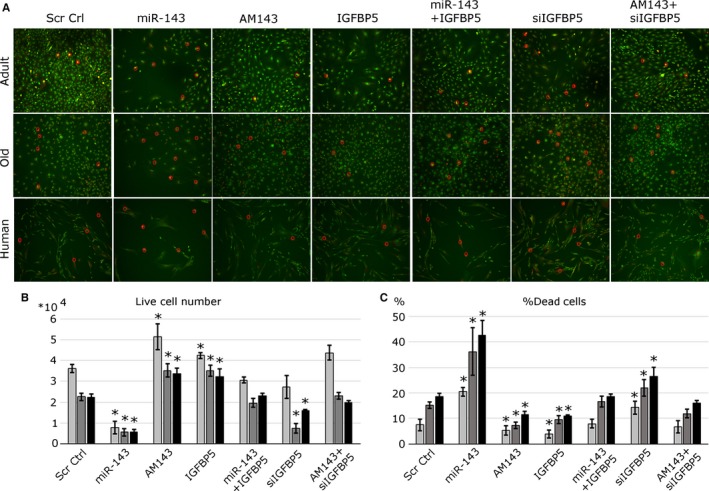
miR‐143 and IGFBP5 affect myoblast viability. Myoblasts isolated from the adult and old mice or adult human muscle were transfected with miR‐143 mimic, inhibitor (AM143), IGFBP5 overexpression construct and/or siRNA against Igfbp5 and cultured in low serum media. (A) Cell viability was established via live/dead staining (green – live cells, yellow/red – cells undergoing apoptosis/necrosis); red circles indicate cells undergoing apoptosis/necrosis. Representative images are shown. (B) The total number of live cells was established by MTS assay. (C) Quantification of % of dead cells is shown. Adult, old – mouse myoblasts; human – adult. Error bars show SEM, * – *P* < 0.05 (compared to control), *n* = 3–4.

Effective muscle regeneration depends on satellite cells viability. Human and mouse myoblasts were seeded at low density and transfected with miR‐143 mimic or inhibitor, or Igfbp5 construct or siRNA. MTS assay was used to quantify the number of viable cells (Fig. 5B). Transfection of miR‐143 had a dramatic effect on the number of viable myoblasts (Fig. 5B). This was not associated with cell proliferation as shown by Ki67 immunostaining (Fig. S5). Increased levels of miR‐143 resulted in decreased cell number and increased myoblasts death as compared with control; this was consistent between mouse and human myoblasts (Fig. 5A, C, S5). Inhibition of miR‐143, as well as IGFBP5 overexpression, resulted in significantly increased viability of myoblasts from humans and mice (Fig. 5A, B, C). Moreover, overexpression of IGFBP5 in cells transfected with miR‐143 mimic or silencing of Igfbp5 expression in myoblasts transfected with AM143 rescued the phenotypes associated with miR‐143 overexpression or inhibition, respectively (Fig. 5).

Overall, these data suggest that miR‐143:Igfbp5 interactions primarily regulate the viability of mouse and human myoblasts controlling the number of viable myoblasts available for differentiation.

### miR‐143:Igfbp5 interactions regulate myoblasts senescence

**Figure 6:**
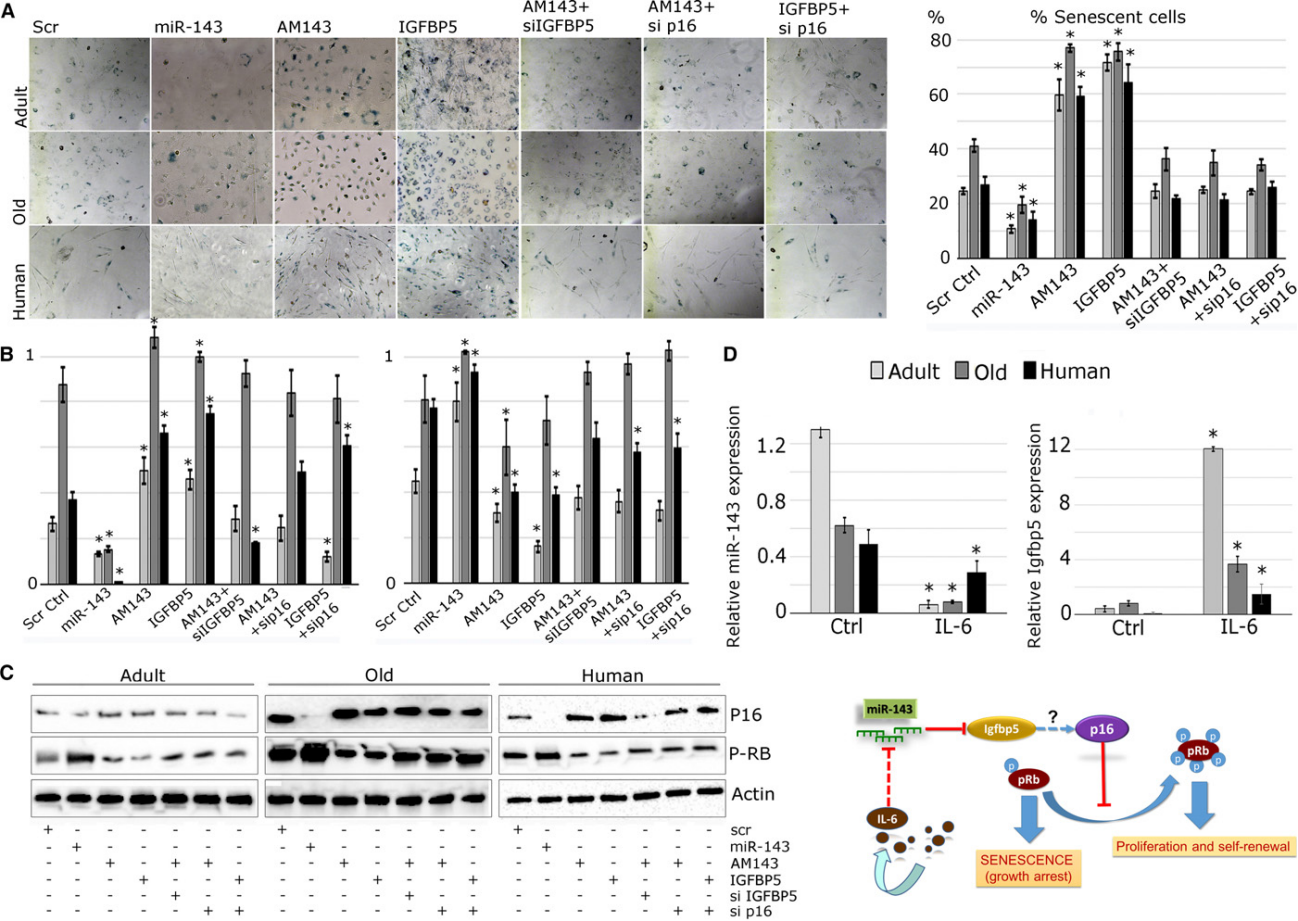
Changes in miR‐143:IGFBP5 interactions are associated with myoblast senescence and affected by IL‐6 levels. (A) Myoblasts isolated from the adult and old mice or human muscle were transfected with miR‐143 mimic, inhibitor (AM143) or IGFBP5 overexpression construct; cell senescence was established via SA‐β‐galactosidase staining (dark blue). Quantification of SA‐β‐galactosidase staining is shown as % of senescent cells. (B) Quantification of Western blots is shown (*n* = 3). (C) Representative Western blot showing the expression of p16 and phosphorylation of Ser608 of RB protein in the myoblast following transfections with miR‐143, AM143, IGFBP5 overexpression construct or siRNA and/or siRNA against p16; adult, old – mouse myoblasts; human – human myoblasts. (D) Primary myoblasts were treated with 0.1 ng mL^−1^ IL‐6. qPCR showing decreased expression of miR‐143 and increased expression of its targets gene, Igfbp5, following IL‐6 treatment. (E) A schematic representation of the role of miR‐143:Igfbp5 interaction in the satellite cells during aging. Expression relative to Rnu‐6 (miR‐143) or β‐2‐microglobulin (Igfbp5) is shown. Error bars show SEM, * – *P* < 0.05 (Student's *t*‐test), *n* = 3–4.

We have shown that IGFBP5 overexpression preserved the viability of myoblasts (Fig. 5); however, it was moderately associated with more efficient fusion into myotubes. Because more unfused myoblasts were observed following the overexpression of IGFBP5, we investigated miR‐143:Igfbp5 regulation of cellular senescence. We stained transfected myoblasts for senescence‐associated‐β‐galactosidase (SA‐β‐gal; Fig. 6). Inhibition of miR‐143 or IGFBP5 overexpression in myoblasts from both mice and human resulted in the detection of more senescent cells (Fig. 6A, C). This was related with increased p16^INK4a^ expression and reduced phosphorylation of RB (Ser608) (Fig. 6B). miR‐143 overexpression resulted in reduced p16^INK4A^ protein expression and increased RB phosphorylation (Fig. 6B–D). Moreover, silencing of p16 ^INK4A^ expression in myoblasts transfected with miR‐143 inhibitor or IGFBP5 overexpression vector largely prevented the increase in senescent cell number, suggesting that miR‐143:Igfbp5 interactions may regulate cell senescence via regulating p16 expression (Fig. 6A–C). These data suggest that decreased levels of miR‐143 and increased levels of its target gene, Igfbp5, protect the myoblasts from atrophy/apoptosis at the cost of their senescence, affecting their myogenic potential.

### miR‐143 and Igfbp5 expression in primary myoblasts is regulated by IL‐6

Having established the role of miR‐143:Igfbp5 interactions in myoblasts, the factors leading to disrupted expression of miR‐143 and Igfbp5 during aging were investigated. Primary myoblasts were treated with IL‐6, a cytokine with suggested role in aging (Ershler *et al*., 1993). IL‐6 treatment of myoblasts resulted in decreased expression of miR‐143 (Fig. 6D) and increased expression of Igfbp5 (Fig. 6D) in the myoblast from humans and mice compared with mock‐treated controls. These data suggest that changes in circulatory factors, such as IL‐6, during aging may influence skeletal muscle regeneration by regulating the expression of microRNAs and their target gene(s) in satellite cells (Fig. 6E).

## Discussion

The molecular mechanisms responsible for age‐related decline in satellite cell function and muscle regeneration remain elusive. Transcriptional networks altered with aging have been well characterized (Giresi *et al*., 2005); however, whether global changes in the expression of genes related to muscle regeneration play a causative or compensatory role in age‐related defective muscle regeneration remains an unanswered question.

miRs are predicted to regulate 2/3 of the human genome, and they are therefore likely to play a major role during aging‐related decline in muscle regeneration. Changes in miRNA expression in muscle with age have been described in rodents and humans (Drummond *et al*., 2008; Kim *et al*., 2014); however, the functional relevance of this is not yet understood.

miR‐143 has been shown to regulate the expression of MyoD in myoblasts (Chen *et al*., 2014). In other tissues, miR‐143 impairs glucose metabolism through the induction of insulin resistance (Jordan *et al*., 2011), regulates the contractility and maintenance of smooth muscle myoblasts (Elia *et al*., 2009) and controls vascular remodelling following injury (Xin *et al*., 2009) and intestinal epithelial regeneration (Chivukula *et al*., 2014).

Our results show that miR‐143 expression is decreased in the satellite cells with age and this is associated with the increased expression of its target, Igfbp5, at both transcript and protein levels.

We show that miR‐143 directly regulates Igfbp5 expression in mouse and human myoblasts (Fig. 2) and that miR‐143:Igfbp5 interactions are disrupted in the satellite cells and during muscle regeneration *in vitro* with age (Fig. 1). For the first time, we demonstrate that miR‐143 regulates myogenesis *in vitro* (Fig. 3). Moreover, we show that miR‐143:Igfbp5 regulates viability and senescence of human and mouse myoblasts (Figs 4 and 5) and therefore affects their myogenic potential (Figs 3, 4, 5, 6). Importantly, our data show that miR‐143:Igfbp5 interactions are conserved between mouse and humans.

Our data reveal that IGFBP5 overexpression resulted in more pronounced phenotype in the myoblasts from the old mice (Figs 4, 5, 6). We propose that this may be a result of age‐related intracellular changes, such as differential expression of IGFBP5‐responsive factors, for example IGF receptors, specifically Igf1r, expression of which is upregulated in the myoblasts from the old mice and humans (Fig. S2C). We have validated Igf1r as miR‐143 target (Fig. S6) and propose that Igf1r is an important mediator of miR‐143 regulation of myogenesis, consistent with previously described role of Igf1r (Duan *et al*., 2010).

IGFBP5 acts both via and independently of IGF signalling pathway (Duan *et al*., 2010), and both negative and positive effects of IGFBP5 on myogenic differentiation have been shown (James *et al*., 1996; Cobb *et al*., 2004; Salih *et al*., 2004; Ren *et al*., 2008). Despite the lack of full understanding of IGFBP5 role in myogenesis, potentially related to different cell types and methodologies used, the role of IGFBP5 in promoting myoblast survival independently of IGFs has been clearly demonstrated (Cobb *et al*., 2004). The role of Igfbp5 in cellular senescence has also been previously shown (Kim *et al*., 2007).

We propose that miR‐143 exerts its function during muscle regeneration through regulating Igfbp5 expression by mainly affecting myoblast viability (Figs 4 and 5) and therefore regulating the number of myoblasts ‘available’ to differentiate. However, our data suggest that human and mouse myoblasts overexpressing Igfbp5, even though more viable, may not be fully functional due to their cellular senescence (Figs 5 and 6). Our data suggest that miR‐143 regulates myoblasts viability by controlling Igfbp5 expression (Figs 2 and 5). As the levels of Igfbp5 expression alone have a moderate effect on myogenesis, we propose that the balance between miR‐143 interactions with Igfbp5 as well as Igf1r regulates myogenic differentiation. Our data clearly demonstrate that the inhibition of miR‐143 or IGFBP5 overexpression results in an increased number of senescent cells and this is most likely regulated, to a large degree, in a p16‐dependent manner as silencing of the expression of p16 prevented the increase in the number of senescent cells following IGFBP5 or AM143 treatment (Fig. 6). We therefore hypothesize that age‐related decrease in miR‐143 expression and concomitant upregulation of its target, Igfbp5, may be a failed attempt of the cells to compensate for age‐related changes in the satellite cells as despite the positive effect of miR‐143 downregulation on cell viability, the concomitant upregulation of IGFBP5 expression may be inducing cellular senescence. This is keeping with previous data showing that such compensatory mechanisms exist. For example, increased expression of miR‐206 in the muscle of ALS mouse model has been shown to compensate the neuromuscular interactions (Williams *et al*., 2009).

Interestingly, increased expression of Igfbp5 and other Igfbps in the muscle has been demonstrated in other than aging scenarios, for example in diabetes also associated with changes in IL‐6 levels (Sreekumar *et al*., 2002). miR‐143 expression is, however, not affected in this model. Based on the recent data by Soares *et al*. (2014), we hypothesize that microRNA‐143 function, as well as Igfbp5 regulation, is context dependent; specifically, miR‐143:Igfbp5 interactions in the satellite cells during aging are likely to be among the key interactions regulating the balance between cell viability and senescence during aging, whereas in other scenarios, such as diabetes or cachexia, Igfbp5 genes may be regulated by a different set of microRNAs.

Finally, we show that IL‐6 controls the expression of both miR‐143 and Igfbp5 in humans and mouse myoblasts *in vitro*. Aging‐related increase in levels of circulatory IL‐6 has been previously reported in humans and mice, and IL‐6 has been proposed as one of the factors contributing to age‐related decline in organism function (Washington *et al*., 2011). The role of IL‐6 in cellular senescence has also been demonstrated (Kojima *et al*., 2013). It remains to be established whether the effects of IL‐6 on miR‐143:Igfbp5 interactions are maintained *in vivo* and whether these indeed contribute to defective muscle regeneration during aging.

In conclusion, our study demonstrated that miR‐143:Igfbp5 interactions play an important role in human and mouse myoblasts viability, senescence and therefore myogenic potential effectively controlling myogenesis *in vitro* and potentially muscle regeneration. We propose that the changes in miR‐143:Igfbp5 interactions in the satellite cells during aging may act as a compensatory mechanism to improve satellite cell function. It remains to be established whether the same mechanisms are maintained *in vivo*. Whether these changes, when induced at the correct time during lifespan, may override the effects of environmental changes, such as elevated levels of circulating IL‐6, and correct the regenerative potential of satellite cells *in vivo* also remains to be shown through a better understanding of mechanisms underlying age‐related defective muscle regeneration; we are opening novel opportunities for more efficient interventions aiming at improved muscle function with age.

## Experimental procedures

For the primers and reagents use, please refer to Tables S1 and S2.

### Mice

The study was performed using male wild‐type C57Bl/6 mice (adult: 6 months old; old – 24 months old). Mice were obtained from Charles River (Margate). All mice were maintained under specific‐pathogen free conditions and fed *ad libitum* a standard chow and maintained under barrier on a 12‐h light/dark cycle. For muscle regeneration, the right tibialis anterior muscle was injured by intramuscular injection of barium chloride (1.2% in saline). For tissue collection, mice were culled by cervical dislocation. The tissues were immediately excised, frozen and stored at −80 °C. Experiments were performed in accordance with UK Home Office guidelines under the UK Animals (Scientific Procedures) Act 1986 and received ethical approval from the University of Liverpool Animal Welfare and Ethical Review Body (AWERB). For each experiment, *n* = 3‐6 biological replicates were used, each biological replicate from *n* = 1 to 2 mice.

### Isolation of primary myoblasts from human skeletal muscle

Experiments were approved by the University of Liverpool, University Hospital Aintree Hospital and Wales Research Ethical Committee 6 and were performed according to good practice guidance. Human skeletal muscle samples (extensor digitorum brevis, tibialis anterior or abductor halluces) derived from foot surgeries of four female patients (adult −30 ± 2.8 years old; older −69 ± 5 years old; BMI < 25) were provided from University Hospital Aintree, Liverpool (UK). Tissue was digested with 1.5 U mL^−1^ collagenase D, 2.4 U mL^−1^ dispase II and 2.5 mm CaCl_2_. Digested muscle was plated on surfaces covered with 10 μg mL^−1^ laminin and incubated with F‐12K media complemented with 20% FBS, 10% horse serum, 2.5 ng/ml FGF‐basic (Recombinant Human FGF‐basic. Peprotech; Ref.: 100‐18B), 1% l‐glutamine and 1% penicillin/streptomycin at 37 °C and 5% CO_2_. Primary myoblasts were grown in DMEM supplemented with 20% FBS, 10% horse serum, 1% l‐glutamine and 1% penicillin/streptomycin.

### Isolation of primary myoblasts from mouse skeletal muscle

Primary myoblasts from the adult (7 months old) and old (24 months old) mice were prepared from EDL muscles following single fibre isolation as previously described (Wang *et al*., 2008; Cheung *et al*., 2012). These myoblasts retained the expression patterns of miRNAs.

### Satellite cells isolation

Satellite cells were isolated using FACS as previously described (Yi & Rossi, 2011). Briefly, skeletal muscle isolated from the hind limbs of two male mice per sorting was treated with 1.5 U mL^−1^ collagenase D, 2.4 U mL^−1^ dispase II and 2.5 mm CaCl_2._ Satellite cells were sorted as α‐7 integrin+, Sca‐1−, CD45−, CD31−. Doublets and haematopoietic and endothelial cells (CD45^+^ and CD31^+^) were excluded from the sorting gates. A pure population of satellite cells negative for Sca1 and highly positive for α‐7 integrin was isolated (CD45^−^, CD31^−^, Sca1^−^, α‐7 integrin^+^). Anti‐α7‐integrin was a gift from Dr. Aldorada Pisconti, University of Liverpool.

### *In vitro* regeneration model

*In vitro* regeneration protocol, where the satellite cells located on isolated myofibres become activated, proliferate, migrate and differentiate, was used to study miR‐143:Igfbp5 interactions (Bischoff, 1986; Pasut *et al*., 2013). Single fibres from EDL muscle from the mice were isolated using collagenase I (Pasut *et al*., 2013) and plated in matrigel‐covered wells (12‐well dishes). The cultures were maintained in DMEM supplemented with 20% foetal bovine serum, 10% horse serum and 1% penicillin/streptomycin. The formation of new myotubes was assessed 10 days following myofibre isolation.

### Cell culture

Primary myoblasts were cultured as described above. To induce myogenic differentiation, 90% confluent cells were cultured in DMEM supplemented with 5% horse serum and 1% penicillin/streptomycin (DM). Myoblast differentiation was assessed 5 days following the switch to DM by immunostaining for myosin heavy chain (Goljanek‐Whysall *et al*., 2012a). To study proliferation and viability, cells were switched to DM once 50% confluent. IL‐6 treatment was performed for 5 days using 0.2 ng mL^−1^ concentration of mouse or human IL‐6. Images were analysed using imagej, loci, university of wisconsin, madison, usa. For morphological analysis, measurement of myotube area was assessed as described (Goljanek‐Whysall *et al*., 2012a).

### Transfections

Myoblasts were transfected with 100 nm miRNA‐143‐3p or anti‐miR or 1 μg Igfbp5 overexpression vector that did not contain 3′UTR (Addgene, deposited by Johnson lab, University of California, San Francisco, CA, USA, Washington University), using Lipofectamine 2000^™^, Life Technologies, Paisley, UK (Goljanek‐Whysall *et al*., 2012a). Mock‐transfected cells served as controls unless otherwise stated. Transfection efficiency was 40–70%, depending on the molecule transfected (Goljanek‐Whysall *et al*., 2012a).

### Real‐Time PCR and Western blotting

RNA isolation and quantitative real‐time PCR were performed using standard methods (Goljanek‐Whysall *et al*., 2014). cDNA synthesis (mRNA) was performed using 500 ng RNA and SuperScript II, and cDNA synthesis (microRNA) was performed using 100 ng RNA and miRscript RT kit II according to the manufacturer's protocol. qPCR analysis was performed using miRScript SybrGreen Mastermix or sso‐Advanced SybrGreen Mastermix in a 20‐μL reaction mixture. Expression relative to β‐2 microglobulin and/or 18S (mRNA) or Rnu‐6 and/or Snord‐61 (microRNA) was calculated using delta delta *C*_t_ method. Data distribution was assessed using Mann–Whitney *U* test, and *P*‐value was calculated using unpaired two‐tailed Student's *t*‐test. Protein lysis and Western blots were performed as described (Goljanek‐Whysall *et al*., 2014).

### *In vitro* miRNA target validation

miR‐143‐3p targets were predicted using TargetScan v.6.2. 3′UTR regions with wild‐type or mutated miR‐143 target site, were synthesized using GeneArt service (Invitrogen, Paisley, United Kingdom) and cloned into a GFP TOPO vector (Invitrogen). Mouse myoblasts were cultured in 6‐well plates and transfected using Lipofectamine 2000^™^ with WT or mutant sensor (1000 ng), with or without miR‐143 or miR‐24 mimic (50 nm) (Goljanek‐Whysall *et al*., 2012a). Transfection with empty GFP vector was used as a negative control. Each experiment was carried out 3× using independent plasmid preparations. Protein was extracted after 48 h, and GFP expression was analysed by Western blotting.

### MTS assay and live/dead staining of human and mouse myoblasts

Myoblast proliferation was assessed by Ki67 staining and MTS assay (Zhang *et al*., 2012). Briefly, cells were transfected as indicated and the proportion of Ki67‐+ cells was assessed 48 h later (Goljanek‐Whysall *et al*., 2012a). Myoblast death was established by live/dead staining. Briefly, cells were washed in PBS and stained with acridine orange/ethidium bromide: PBS (1:1000); images were taken 5–60 min following the staining.

### Staining of primary myoblasts culture to determine senescence

Senescence staining of primary myoblasts was performed 7 days after transfections using Senescence β‐Galactosidase Staining Kit (9860; Cell Signalling Technology 3 Trask Lane, Danvers, MA 01923) according to the manufacturer's protocol. Pictures were captured with Zeiss Axiovert 200M Research Inverted Microscope at 10× magnification power. Senescence staining was quantified as described in Acosta *et al*. (2008).

## Author contributions

KW, AS and RM performed the experiments; AMcA provided expertise and advice in techniques used; AM provided human muscle samples. All authors contributed to preparing the manuscript.

## Conflict of interests

The authors declare no conflict of interest regarding the publication of this article.

## Funding

This work is supported by the Biotechnology and Biological Sciences Research Council (BBSRC; BB/L021668/1), the MRC and Arthritis Research UK as part of the MRC – Arthritis Research UK Centre for Integrated Research into Musculoskeletal Ageing (CIMA) and the Wellcome Trust Institutional Strategic Support Fund (097826/Z/11/A).

## Supporting information


**Fig. S1** Satellite cell purification by fluorescence‐activated cell sorting (FACS).

**Fig. S2** Expression of miR‐143 and miR‐143 predicted target genes in muscle and myoblasts during aging.

**Fig. S3** miR‐143 and IGFBP5 expression can be effectively modulated in myoblasts.

**Fig. S4** miR‐143 negatively regulates terminal differentiation of mouse myoblasts.

**Fig. S5** miR‐143:Igfbp5 interactions have limited effect on myoblasts proliferation.

**Fig. S6** miR‐143 represses expression of Igf1r in primary myoblasts.

**Table S1** A list of real‐time PCR primers used.
**Table S2** A list of reagents used.
