# Supplementary figures and images for: Age‐related changes in miR‐143‐3p:Igfbp5 interactions affect muscle regeneration
Source: Aging Cell. 2016 Jan 13;15(2):361–9. doi: 10.1111/acel.12442 (PMC4783349; doi:10.1111/acel.12442)

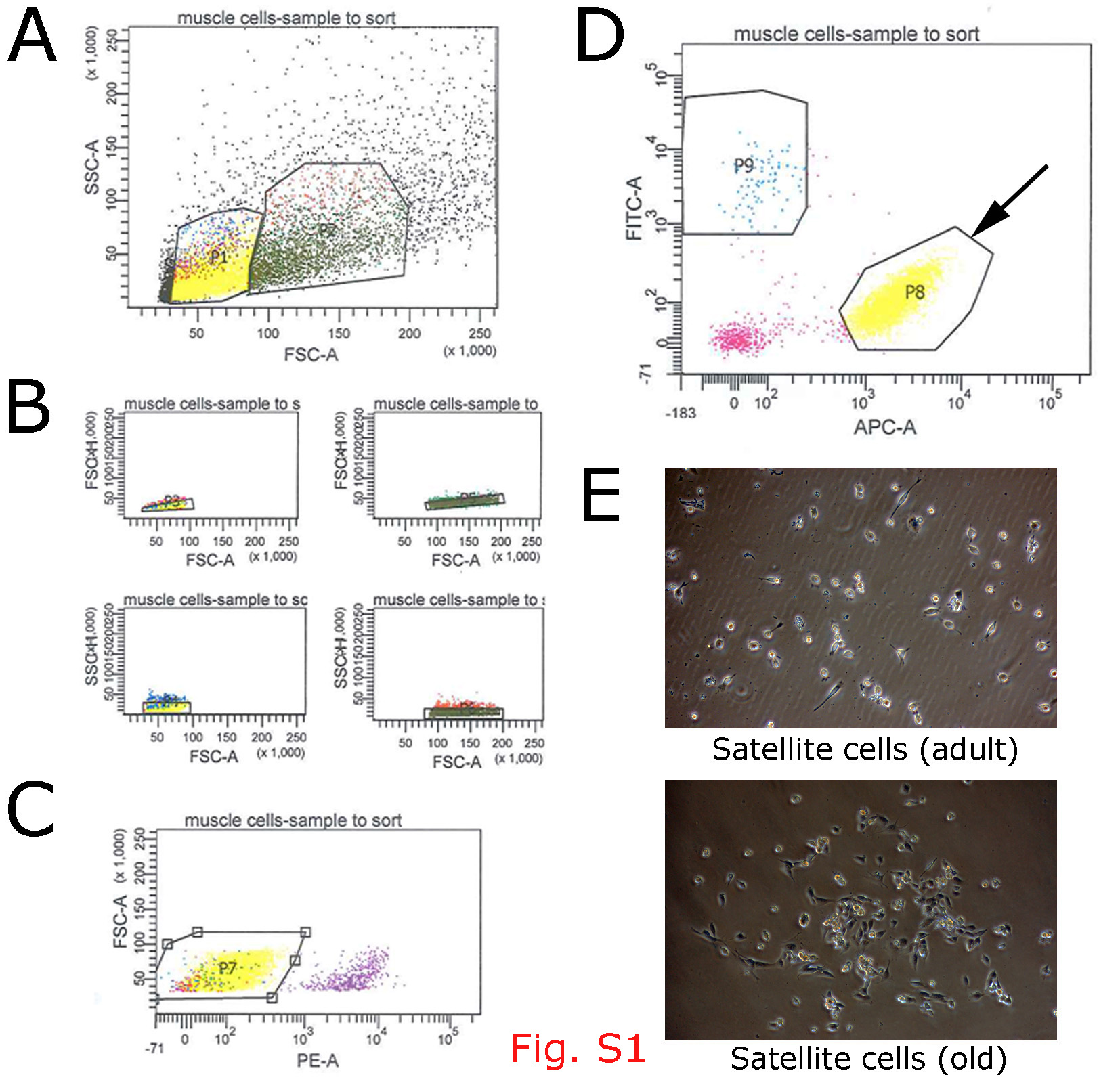

Supplement: Supplementary file 1 — Fig. S1 Satellite cell purification by fluorescence‐activated cell sorting (FACS). [file ACEL-15-361-s001.jpg]

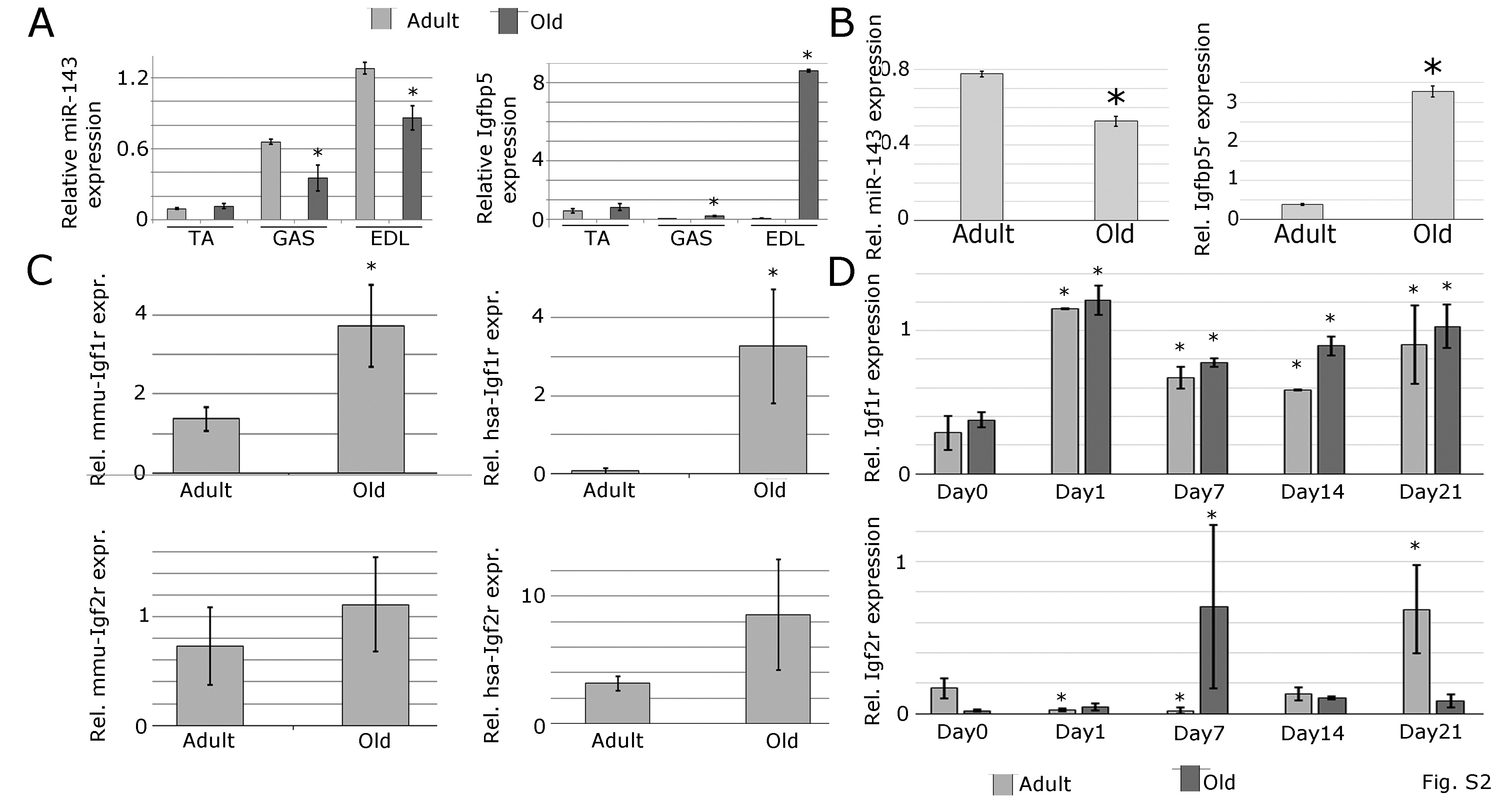

Supplement: Supplementary file 2 — Fig. S2 Expression of miR‐143 and miR‐143 predicted target genes in muscle and myoblasts during aging. [file ACEL-15-361-s002.jpg]

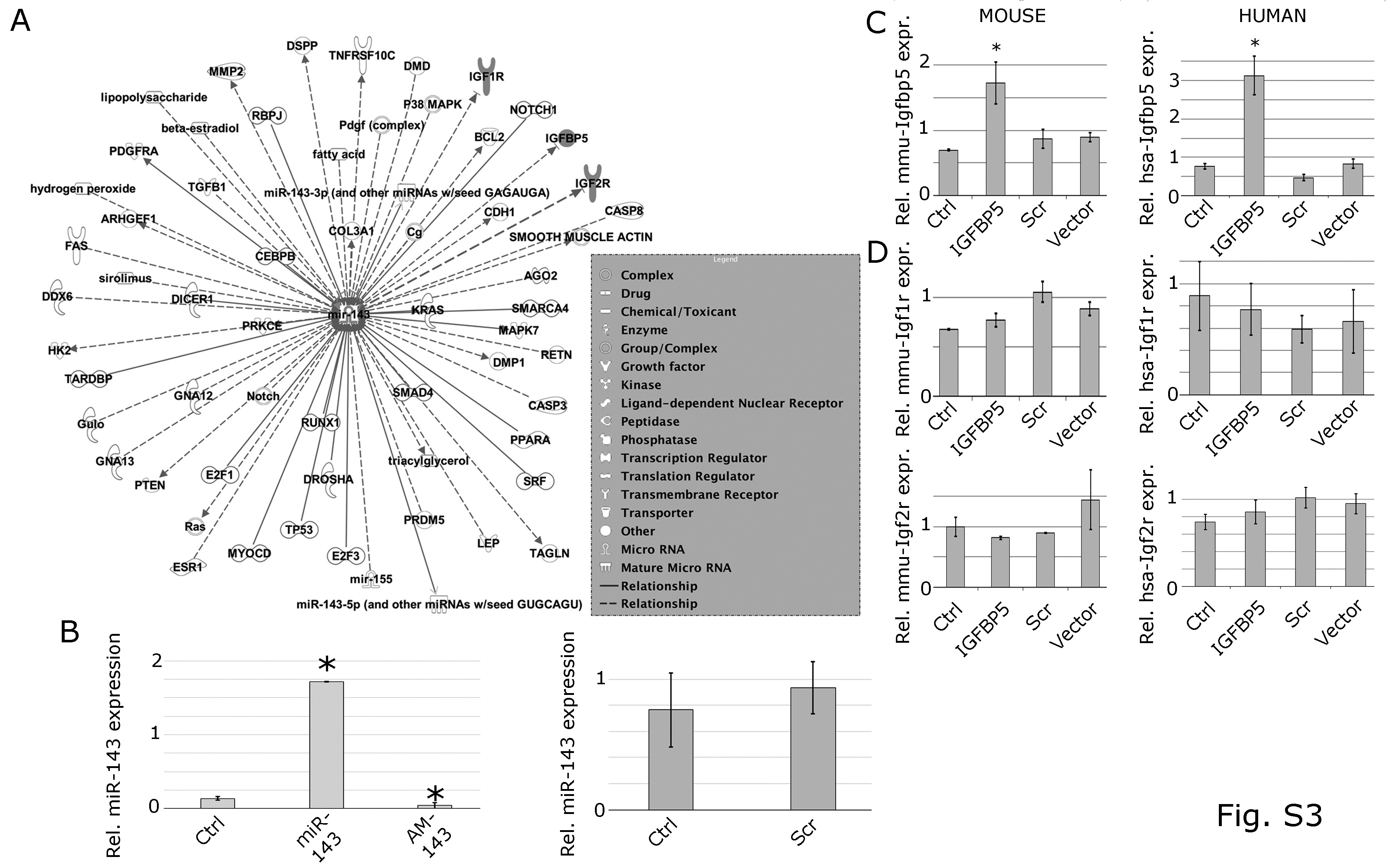

Supplement: Supplementary file 3 — Fig. S3 miR‐143 and IGFBP5 expression can be effectively modulated in myoblasts. [file ACEL-15-361-s003.jpg]

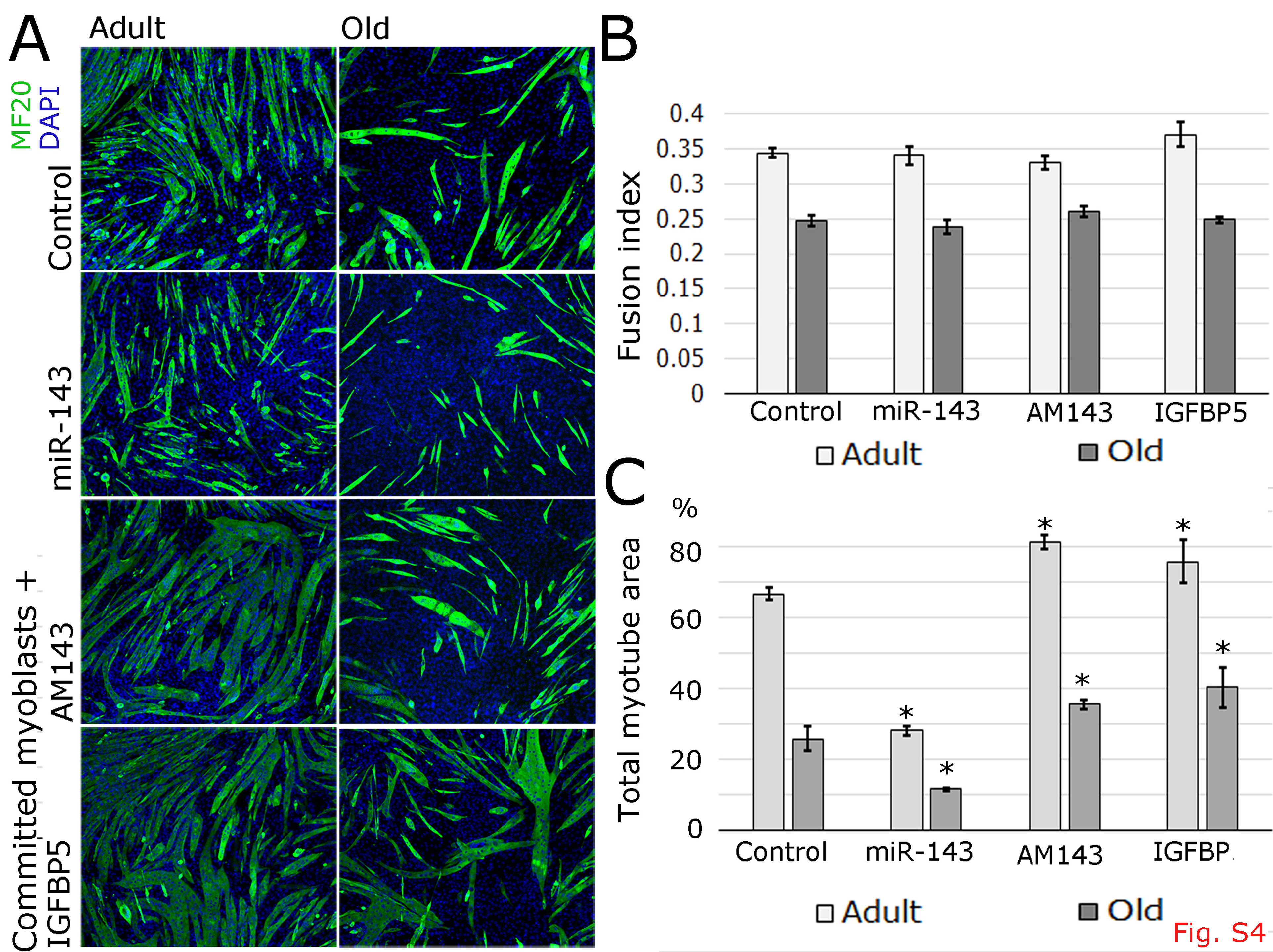

Supplement: Supplementary file 4 — Fig. S4 miR‐143 negatively regulates terminal differentiation of mouse myoblasts. [file ACEL-15-361-s004.jpg]

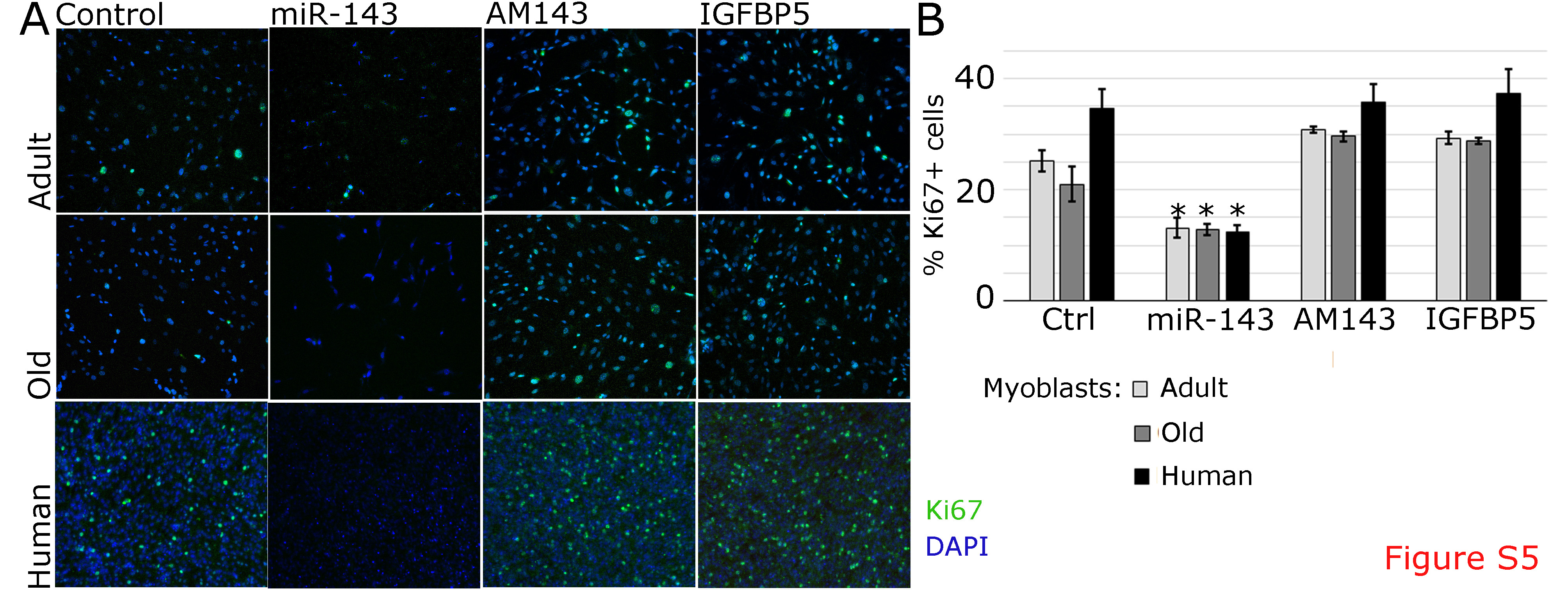

Supplement: Supplementary file 5 — Fig. S5 miR‐143:Igfbp5 interactions have limited effect on myoblasts proliferation. [file ACEL-15-361-s005.jpg]

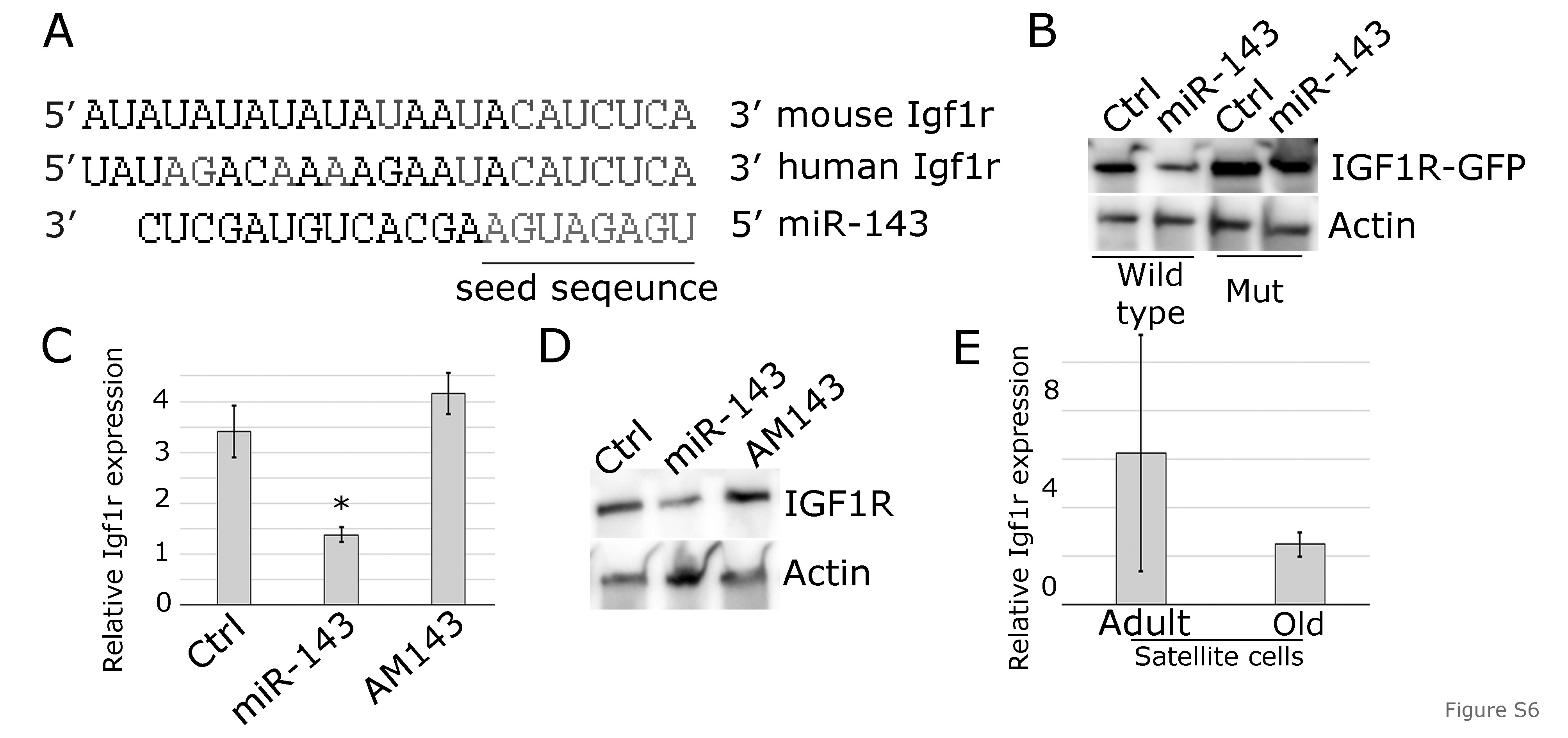

Supplement: Supplementary file 6 — Fig. S6 miR‐143 represses expression of Igf1r in primary myoblasts. [file ACEL-15-361-s006.jpg]
