# Supplementary material for: Age‐related changes in miR‐143‐3p:Igfbp5 interactions affect muscle regeneration
Source: Aging Cell. 2016 Jan 13;15(2):361–9. doi: 10.1111/acel.12442 (PMC4783349; doi:10.1111/acel.12442)
Supplement: Supplementary file 7 — Table S1 A list of real‐time PCR primers used. Table S2 A list of reagents used. [file ACEL-15-361-s007.docx]

| **Gene** | **Forward primer** | **Reverse primer** |
| --- | --- | --- |
| Igfpb5 | TACGGCGAGCAAACCAAGAT | CAGGGGCCTTGTTCGGATTC |
| Igf1r | CTGATGTCTGGTCCTTCGGG | TGATGCTGCCGATGATCTCC |
| P16 | TGGTCACTGTGAGGATTCAGC | GTTGCCCATCATCATCACCTGG |
| Beta-2-microglobulin | GGAGAATGGGAAGCCGAACA | TCTCGATCCCAGTAGACGGT |
| 18S | CGGCTACCACATCCAAGGAAGG | CCCGCTCCCAAGATCCAACTAC |

**Table S1.**

| **Product** | **Product number** | **Source** |
| --- | --- | --- |
| **Muscle digestion** |  |  |
| Collagenase D | 11088866001 | Roche |
| Dispase II | D4693 | Sigma-Aldrich |
| Calcium chloride | 449709 | Sigma-Aldrich |
| Laminin | L2020 | Sigma-Aldrich |
| F12-K media | 21127-022 | Gibco® |
| FGF-basic | 100-18B | Peprotech |
| Matrigel | 356234 | BD Biosciences |
| DMEM | D5671 | Sigma-Aldrich |
| **Antibodies** |  |  |
| CD31 antibody | 340297 | BD Biosciences |
| CD45 antibody | 562420 | BD Biosciences |
| Sca-1 antibody | 558162 | BD Biosciences |
| MF20 antibody | MF20 | Developmental Studies Hybridoma Bank |
| IGFBP5 antibody | Ab86463 | Abcam |
| Actin antibody | Ab185058 | Abcam |
| IGF1R antibody | Ab131476 | Abcam |
| phosphor antibody | 2181s | Cell Signalling |
| GFP antibody | 10362 | Life Technologies |
| Cleaved Caspase-3 antibody | Ab77973 | Abcam |
| P16 antibody | Ab51243 | Abcam |
| Ki67 antibody | Ab15580 | Abcam |
| **Transfections, cell culture** |  |  |
| Mouse IL-6 | PMC0063 | Life Technologies |
| Human IL-6 | PHC0065 | Life Technologies |
| miR-143 mimic | MSY0000247 | Qiagen |
| miR-143 inhibitor (antimiR) | MIN0000247 | Qiagen |
| mR-24 mimic | MSY0000219 | Qiagen |
| Lipofectamine2000 | 11668027 | Life Technologies |
| SA-b-Galactosidase staining kit | 9860 | Cell Signalling Technology |
| GFP TOPO | K4810-01 | Life Technologies |
| siRNA against human Igfbp5 | 45935 | Life Technologies |
| siRNA against mouse Igfbp5 | 159129 | Life Technologies |
| siRNA against human p16 | 118858 | Life Technologies |
| siRNA against mouse p16 | 262865 | Life Technologies |
| **Real-Time PCR** |  |  |
| Superscript II | 18064 | Life Technologies |
| miRScript RT II | 218161 | Qiagen |
| miRScript SybrGreen | 218073 | Qiagen |
| Sso-Advanced SybrGreen | 172-5270 | Biorad |
| miR-143 qPCR primer | MS00001617 | Qiagen |
| RNU-6 qPCR primer | MS00033740 | Qiagen |
| Snord-61 qPCR primer | MS00033705 | Qiagen |

**Table S2.**
